# P-cadherin Overexpression Induces Partial Epithelial-to-Mesenchymal Transition and Confers Chemoresistance in Dysplastic Oral Epithelial Cells

**DOI:** 10.7150/jca.135818

**Published:** 2026-10-16

**Authors:** Aaron Suresh Mody, Christine Paulette Neal, Kristen Kim Drinkwater, Cassandra Lynn Decker, Kathryn Rene Lawson

**Affiliations:** Department of Biochemistry and Molecular Genetics, College of Graduate Studies, Midwestern University, Department of Biochemistry, Glendale, AZ 85308, USA.

**Keywords:** P-cadherin, oral dysplasia, epithelial-to-mesenchymal transition, chemoresistance, epidermal growth factor receptor, mucin-1

## Abstract

Chemoresistance is a major barrier to the successful treatment of oral squamous cell carcinoma. The dysplasia stage of oral tumor progression is associated with increased levels of antiapoptotic mediators as well as the cell adhesion protein placental cadherin (P-cadherin). The mechanisms by which P-cadherin may contribute to tumor progression in dysplastic oral epithelia remain unclear. In the present study, a dysplastic oral keratinocyte (DOK) cell line was utilized as a model system to investigate the effects of P-cadherin overexpression on cellular behavior and survival signaling. Compared with the controls, DOK cells that overexpressed P-cadherin displayed a mesenchymal morphology and increased levels of the chemoresistance modulator, mucin-1. P-cadherin overexpression resulted in a 2-fold decrease in apoptosis levels in response to treatment with the chemotherapeutic agent 5-fluorouracil. In the same drug-treated cells, P-cadherin overexpression increased phosphorylation of the survival kinases Akt and mitogen-activated protein kinase, as well as levels of focal adhesion kinase. The morphology changes and chemoresistance displayed by P-cadherin-overexpressing cells were consistent with an epithelial-to-mesenchymal transition. This transition may be partial, however, as P-cadherin-overexpressing cells retained expression of epithelial cadherin and exhibited a 2-fold decrease in motility. Taken together, these data suggest that during oral dysplasia, P-cadherin may continue to participate in cell-to-cell contacts while also modulating chemoresistance through the activation of survival signaling pathways.

## Introduction

In 2026, an estimated 60,480 Americans will be diagnosed with oral or oropharyngeal cancer. Nearly 13,150 will die from this disease 1. Although primary tumors can be treated with surgical resection and/or radiotherapy 2, one in three patients will experience tumor recurrence following treatment. Patients with oral carcinoma with recurrent tumors display an average 5-year survival rate of 30% 3. The effectiveness of surgical and radiologic treatments for oral cancer is hampered by oral field cancerization, a phenomenon in which cells within premalignant patches of oral epithelia give rise to new primary or secondary tumors 4. Systemic treatment with chemotherapeutic agents has been utilized to reduce recurrence, however, chemoresistance is a common and significant barrier to successful treatment outcomes 5. Alterations in both mitogenic and apoptotic signaling have been described in dysplastic oral epithelia 5-7, suggesting that the molecular basis for drug resistance may be established during the early stages of oral tumor progression. An improved understanding of this process is essential for improving the efficacy of treatment modalities and preventing tumor recurrence in high-risk individuals.

Recent research suggests that the chemoresistance observed in dysplastic oral epithelia may reflect a form of cellular reprogramming known as the epithelial-to-mesenchymal transition (EMT) 6, 8. During EMT, cells acquire mesenchymal characteristics, including altered morphology, increased migration, and decreased synthesis of cell adhesion molecules. The transcription networks activated during EMT were shown to increase cell proliferation and resistance to both senescence and apoptosis 9. A hallmark of EMT is altered expression of the cadherins, a family of calcium-dependent transmembrane proteins that mediate cell-cell adhesion. In healthy oral epithelia, epithelial cadherin (E-cadherin) maintains adhesion in the basal and suprabasal layers, but E-cadherin levels become reduced or absent in severe dysplasia and carcinomas 6 (Fig. 1). A well-characterized feature of EMT is the 'cadherin switch', in which downregulation of E-cadherin is accompanied by ectopic expression of neural cadherin (N-cadherin) 10. Increased levels of N-cadherin were observed in oral carcinomas and correlated with poor prognosis 11.

Like E-cadherin, P-cadherin also mediates cell adhesion in the basal layer of healthy oral epithelia. In mild to severe oral dysplasia, levels of membrane P-cadherin were shown to increase in both the basal and parabasal layers compared with healthy oral epithelia (Fig. 1) 12, 13. In late-stage oral carcinomas, P-cadherin expression is reduced or absent (Fig. 1) 14, 15. In breast carcinoma models, transient P-cadherin expression has been shown to promote an intermediate or 'metastable' phenotype, in which cells have acquired mesenchymal characteristics yet retained epithelial traits 16. Little is known about the consequences of P-cadherin overexpression during oral dysplasia, as most mechanistic studies utilized oral squamous carcinoma cell models 14, 17.

Although P-cadherin upregulation has been documented in oral dysplasia, few studies have examined the functional contributions of this increase to tumor progression. We hypothesized that increased P-cadherin in dysplastic keratinocytes would induce features of EMT and confer resistance to 5-fluorouracil. To test this, we overexpressed P-cadherin in dysplastic human oral keratinocytes and assessed effects on EMT, chemoresistance, and related signaling pathways.

## Materials and Methods

*Cell culture*. Dysplastic oral keratinocytes (DOK) were purchased from the European Collection of Cell Cultures, via Sigma-Aldrich (Merck KGaA). The DOK line was derived from a dysplastic oral lesion found in the tongue of a heavy smoker 18, 19. Keratin profiling of these cells demonstrated keratin expression patterns that matched those expressed by the dysplastic oral tissue from which they originated. These data confirm the epithelial and keratinocyte nature of the cell line 18. The DOK line is naturally immortalized and does not form tumors in nude mice. It has been characterized as mildly to moderately dysplastic 18, 20. DOK cells were maintained in high glucose Dulbecco's modified Eagle's medium (DMEM) supplemented with 10% fetal bovine serum (Hyclone; Cytiva).

Retroviral transduction and selection of DOK cells to produce the vector-only control, P-cadherin overexpressing and N-cadherin-depleted lines were performed as previously described 17, 21. Briefly, cDNA for human P-cadherin was cloned into the retroviral expression vector pLZRS-MS-Neo 22. This plasmid directs synthesis of single mRNA that consists of a cDNA for P-cadherin followed by an internal ribosome entry site that independently directs translation of the *NeoR* neomycin resistance gene. DOK cells that were used as controls were retrovirally transduced to express the same mRNA that encoded *NeoR* but did not synthesize P-cadherin. For depletion of endogenous N-cadherin transcripts, the pSuper.retro.neo vector was utilized to synthesize a small hairpin RNA against human N-cadherin mRNA in virally-transduced cells 23. This vector also expresses the *NeoR* neomycin resistance gene to enable neomycin selection of infected cells. To construct this small hairpin RNA, the sequence GACTGGATTTCCTGAAGAT and its corresponding antisense sequence ATCTTCAGGAAATCCAGTC were introduced into the pSuper.retro.neo vector according to manufacturer's instructions (Oligoengine, Seattle, WA). Production of amphotropic retrovirus and subsequent infection of DOK cells was performed as previously described 17. All transduced lines were both selected and maintained using 400 µg/mL G418 (Santa Cruz Biotechnology, Inc.).

Primary human oral keratinocytes (HOK) were obtained from ScienCell Research Laboratories (cat. no. 2610). These cells were established from human oral mucosa and cryopreserved at passage 1 before receipt. These cells were found to express keratin 18 and 19, which are characteristic of normal buccal keratinocytes 24. HOK cells were maintained in oral keratinocyte media (ScienCell Research Laboratories; cat. no. 2611). All cell lines were maintained at 37ºC with 5% CO_2_. Product information, including manufacturer, catalog number, and product webpage, for all cell lines and antibodies used in this study is provided in Table S1.

*Microscopy and image acquisition.* Bright-field photography of cell cultures and wounded cell monolayers was performed utilizing an Axiovert 40 inverted microscope (Zeiss AG). Immunofluorescence photography for EGFR/P-cadherin colocalization was performed on DOK cells using a Zeiss ApoTome microscope (Zeiss AG). Image capture and analysis were performed utilizing AxioVision 4.8 software (Zeiss AG).

*Western blot analysis.* For measurement of steady-state protein levels, cells were plated 48 h before harvest. Cells were serum starved 18 h before harvest. For chemotherapeutic studies, DOK cells were plated in the DMEM culture media described above for 48 h. Cells were then rinsed with phosphate-buffered saline (PBS) (cat. no. BP399500, Fisher Scientific) and fresh culture media containing 3 µM 5-fluorouracil (Sigma-Aldrich) was applied. Cells were harvested for western blot analysis at 24 h after treatment. Adherent cells were washed with cold PBS twice before adding lysis buffer. Cells were lysed with radioimmunoprecipitation assay buffer (150 mM sodium chloride, 1.0% NP-40, 0.5% sodium deoxycholate, 0.1% sodium dodecyl sulfate and 50 mM Tris, pH 8.0) supplemented with HALT protease and phosphatase inhibitor cocktail (Thermo Fisher Scientific, Inc.). Plates were scraped vigorously. Samples were collected via tuberculin syringes and triturated five times into a centrifuge tube. Samples were spun down at 4ºC for 5 min. The supernatant was collected and stored at -80ºC. Protein concentration analysis of lysates was performed using the Pierce BCA Protein Assay Kit (Thermo Fisher Scientific, Inc.). Samples were read on the BioTek Synergy 2 plate reader.

Equal quantities of protein were analyzed by SDS-PAGE and subjected to western immunoblotting. Primary antibodies used in this study included mouse monoclonal antibodies directed against P-cadherin (1:1000; cat. no. 14029; Cell Signaling Technology), E-cadherin (1:1000; cat. no. 14472; Cell Signaling Technology, Inc.), N-cadherin (1:1,000; cat. no. 14215; Cell Signaling Technology, Inc.), β-catenin (1:1000; cat. no. 37447; Cell Signaling Technology, Inc.), p120 (1:2,000; cat. no. 610134; BD Biosciences), β-tubulin (1:500; cat. no. E7; Developmental Studies Hybridoma Bank, University of Iowa) and MUC1 N-terminal subunit (1:1000; cat. no. 4538; Cell Signaling Technology, Inc.). All rabbit-derived antibodies were purchased from Cell Signaling Technology, Inc., diluted 1:1000, and included phospho-epidermal growth factor receptor (EGFR^Y1045^; cat. no. 2237), total epidermal growth factor receptor (EGFR; cat. no. 4267), proliferating cell nuclear antigen (PCNA; cat. no. 13110), phospho-p42/p44-mitogen-activated protein kinase (MAPK^T202/Y204^; cat. no. 4370), total p42/p44-mitogen-activated protein kinase (MAPK; cat. no. 4695), phospho-Akt^S473^ (cat. no. 4060), total Akt (cat. no. 4691), phospho-glycogen synthase kinase 3β (GSK3β^S9^; cat. no. 9323), total glycogen synthase kinase 3β (GSK3β; cat. no. 12456), phospho-focal adhesion kinase (FAK^Y397^; cat. no. 8556), and total focal adhesion kinase (cat. no. 71433). Secondary antibodies used included DyLight 680 goat anti-mouse (cat. no. 5470) and anti-rabbit (cat. no. 5366) antibodies, as well as DyLight 800 anti-mouse (cat. no. 5257) and anti-rabbit (cat. no. 5151) antibodies. All DyLight antibodies were utilized at 1:5,000 dilution and were purchased from Cell Signaling Technology, Inc. Blots were visualized and quantitated on the Odyssey CL-x Imaging System (LI-COR Biosciences) using ImageStudio Lite software (version 5.0; LI-COR Biosciences).

For protein quantitation, western blot membranes were first stained with Revert total protein stain (LI-COR Biosciences) to visualize and quantitate all bands in each lane. ImageStudio software was used to delineate equal areas of analysis for each lane to quantitate total protein fluorescence for each lane. Revert destain was applied before incubation with primary antibodies. Band intensities obtained for each protein of interest were normalized to the total protein stain signal for each lane.

*Immunofluorescence staining.* For immunofluorescence staining, DOK cells growing on glass coverslips were fixed using 10% neutral-buffered formalin (Thermo Fisher Scientific, Inc.) for 30 min and permeabilized with 0.5% Triton X-100 (Thermo Fisher Scientific, Inc.) for 15 min. Coverslips were blocked in 10% goat serum (cat. no. 5425; Cell Signaling Technology, Inc.) for 1 h. For the co-localization experiment in Fig. 6A, immunofluorescence was performed on fixed, blocked cells using a mouse monoclonal antibody against P-cadherin (1:100; cat. no. 14029; Cell Signaling Technology) and an Alexa 594 anti-mouse secondary (1:100; cat. no. A-11005; Thermo Fisher Scientific, Inc.), or an anti-EGFR rabbit polyclonal antibody (1:100; cat. no. 4267; Cell Signaling Technology) and an Alexa Fluor Plus 488 anti-rabbit secondary antibody (1:100; cat. no. A32731; Thermo Fisher Scientific, Inc.). All slips were mounted in mounting media containing 4,6-diamidino-2-phenylindole (DAPI; Vector Laboratories, Inc.) and imaged using an ApoTome fluorescence microscope. AxioVision 4.8 software was used to collect fluorescent images and to create merged images for each image set.

*Motility assays.* For wound healing assays, control and P-cadherin-overexpressing DOK cells were plated in equal numbers into 60 mm dishes that contained 2 mm etched grids (Corning, Inc.). After 24 h, fresh DMEM containing 5% fetal bovine serum was added. After an additional 24 h, confluent monolayers were scratched using 200 µL pipette tips. Grid locations and cells within recorded grid sections were photographed immediately after the scratch and 8 h later. Image J software 25 was used to quantify the pixels within the open (i.e., cell-free) area of each scratch for all photographs. The pixel area of each cell line at 8 h was subtracted from the pixel area at 0 h to calculate the Area of Closure (AoC). Fold change was calculated by dividing the AoC values for each cell line by the mean control AoC; thus, the mean control value equals 1, and its error bar reflects the variation among control replicates. Error bars depict ± standard error of the mean. Values reported represent three independent experiments, each with one plate per cell type (n=3).

*Proliferation analysis*. DOK control cells and P-cadherin-overexpressing cells were rinsed with PBS and dislodged from the cell culture dish using an Accumax dissociation solution (Innovative Cell Technologies, Inc.). Accumax was then neutralized by dilution with an equal volume complete media consisting of DMEM with 10% serum. Cells were counted using a hemocytometer and plated into wells of an 8-well culture dish (BD Falcon; Becton, Dickinson and Company). After 24 h plating, cells were switched to serum-free DMEM for an additional 24 h. At this time, cells were incubated in DMEM containing 10% fetal bovine serum.

The proliferation of these cells was then analyzed using the Click-iT EdU Proliferation Assay (Invitrogen; Thermo Fisher Scientific, Inc.). After 24 h administration of complete media, cells were treated with 10 μM 5-ethynyl-2'-deoxyuridine (EdU) reagent (Invitrogen; Thermo Fisher Scientific, Inc.) diluted in complete media. Following an additional 24 h of EdU treatment, cells were then fixed with 10% neutral-buffered formalin (Thermo Fisher Scientific, Inc.) and permeabilized with 0.2% Triton X-100 (Research Products International Corp.) in PBS. Cells were labeled with the Click-iT reaction cocktail following the manufacturer's instructions. Stained cells were mounted with VectaShield (Vector Laboratories, Inc.) containing DAPI to visualize cell nuclei. Cells were visualized and photographed using the ApoTome fluorescence microscope at x100 magnification. AxioVision software was used to color immunofluorescent images and to create merged images.

Images were analyzed with ImageJ software 25 to determine the percent of EdU-labeled cells in each cell set. The total percent of EdU labeled cells was determined by taking the ratio of the number of cells counted in the 488 channel, which reflected EdU incorporation, and dividing by the number of cells counted in the DAPI channel. Data represent eight independent replicates for each cell line (n=8).

*Morphology analysis.* The transition of cells from cuboidal to spindle-shape that occurs during EMT was previously quantitated by calculating the aspect ratio of individual cells 26. The aspect ratio is a numerical value obtained by dividing the length of the cell at its longest axis by the width of the cell at its shortest axis. Cuboid epithelial cells have similar length and width characteristics and exhibit an aspect ratio of ~1.0. Mesenchymal cells that display a spindle morphology have a longer length but shorter width and often display an increased aspect ratio compared to cuboidal cells 26, 27.

We used this method to quantitate changes in dysplastic oral epithelial cell morphology induced by P-cadherin overexpression. DOK control and P-cadherin-overexpressing cells were plated at equal densities in three separate 60 mm dishes per cell line and grown for 24 h. Cells were photographed at x200 magnification using an Axiovert 40 inverted microscope and a DMC-LX5 digital camera (Panasonic). One photograph was taken of each dish (three per cell line) by another research staff member who was blinded to cell identity. ImageJ software 25 was used to overlay a grid over each photograph. To randomize cell selection, individual cells at every other intersection of grid lines were included for morphology analysis. Measurements of the longest and shortest axis values were taken from 25 cells per photograph (75 cells per line) using the measurement tool in Image J 25. The pixel length of each cell was divided by the pixel width to generate an individual aspect ratio for each cell. Aspect ratios of all cells from each line (n=75) were averaged.

*Apoptosis analysis.* The DOK cells were analyzed using the RealTime-Glo Annexin V Apoptosis and Necrosis Assay (Promega Corporation) according to the manufacturer's instructions. DOK cells (10,000 cells/well) were plated in white, solid-bottom 96-well plates (Greiner Bio-One, cat. no. 655083) in DMEM with 10% fetal bovine serum. After 48 h of plating, an equal volume of culture medium containing 5-fluorouracil was added to each well. This addition yielded final concentrations of 0.0, 0.75, 1.5, 3.0, 6.0, 12.0, and 24.0 µM. Fluorescence and luminescence were analyzed at 0 and 24 h after drug addition on a Cytation 5 plate reader using Gen5 analysis software (both from Biotek Instruments, Inc.). Apoptosis data were expressed as relative luminescence units (RLU) and represent the averages of three independent biological replicates for each timepoint and concentration. Values from untreated (0 µM 5-fluorouracil) wells for each cell line were subtracted from each value.

*Statistical analysis.* Quantitated results are expressed as a mean ± standard error. Mean ± standard deviation values for the data presented in Figs. 2-5 are provided in Supplementary Tables S2-S4. Statistical significance for all experiments was determined by a two-tailed Student's t-test. Formal testing for normality was not performed due to the small sample sizes employed (typically n=3 biological replicates); the assumption of normality is considered reasonable for this type of analysis, consistent with common practice in the field. P≤0.05 was considered to indicate a statistically significant difference. Statistical analyses were performed using Microsoft Excel software (Microsoft Corporation).

*Artificial intelligence tools.* Artificial intelligence tools (Claude, Anthropic) were utilized during manuscript revision to provide editorial feedback on manuscript structure, evaluate clarity of scientific narrative, and assist with proofreading. AI tools did not contribute to the scientific content, experimental design, data interpretation, or conclusions presented in this work. All intellectual contributions and final editorial decisions were made by the authors.

## Results

### Cadherin expression in normal and dysplastic oral keratinocytes

The dysplastic oral keratinocyte (DOK) cell line utilized in this study was previously characterized as mildly to moderately dysplastic. To our knowledge, the cadherin expression profile of these cells had not yet been systematically compared with that of normal oral keratinocytes. We were interested in determining the alterations in cadherin expression in these cells compared with those of primary normal human oral keratinocytes (HOK). It was also of interest to determine the extent to which the changes seen in the DOK line paralleled those observed in immunological analyses of dysplastic oral tissue sections. Western blotting analysis (Fig. 2A) displayed a 4.3-fold increase in P-cadherin in DOK cells compared to the HOK cells (Fig. 2B). A 5.5-fold increase in N-cadherin was also observed (Fig. 2B). A slight but significant increase was also found in levels of the cadherin regulator p120 catenin (Fig. 2C). Levels of E-cadherin and the adherens junction component β-catenin were similar across all cell lines examined. Overall, the levels of cadherins and catenins that we observed in this cell line are consistent with those reported for dysplastic oral epithelia 12, 13. Epidermal growth factor receptor was also found to increase 3.6-fold (Fig. 2C), which is consistent with the upregulation of EGFR observed in dysplastic oral epithelia 28. Similar increases were observed in levels of proliferating cell nuclear antigen (Fig. 2C).

The DOK line that was engineered to overexpress P-cadherin displayed a 2.5-fold increase in P-cadherin levels compared to DOK control cells (Fig. 2B). Overexpression of P-cadherin did not statistically alter levels of any of the other proteins examined in the panel (Fig. 2B and C). This line was subsequently used to examine the effects of increased P-cadherin on EMT characteristics and chemoresistance.

### P-cadherin overexpression induces characteristics of EMT

To assess the effects of P-cadherin on EMT we first quantified morphology changes induced by P-cadherin overexpression (Fig. 3). The change in morphology from a relatively circular, cuboidal epithelial cell to a spindle-shaped mesenchymal cell produces an increase in length compared to the width (Fig. 3A). Thus, the aspect ratio, determined by dividing cell length by width, increases as cells become more mesenchymal in nature. The aspect ratio of the DOK lines measured was visibly different in response to P-cadherin overexpression (Fig. 3B). The average aspect ratio of control DOK cells was 1.3 and the average aspect ratio of P-cadherin overexpressing DOK cells was 1.8 (Fig. 3B). Statistical analysis confirmed that there was a significant increase in aspect ratio with P-cadherin overexpression (n=75). Mucin-1 (MUC1) is a membrane-associated glycoprotein that promotes EMT through the activation of EMT-related transcription factors 29 and is elevated in oral potentially malignant disorders 30. P-cadherin overexpression increased MUC1 protein levels in DOK cells (Fig. 3C).

### P-cadherin overexpression in dysplastic keratinocytes suppresses motility

P-cadherin and MUC1 proteins have been independently associated with increases in cell motility in oral squamous carcinoma cells 17, 31. To extend these studies to a dysplastic model, we utilized a wound-healing assay to compare the migration of control and P-cadherin overexpressing DOK cells (Fig. 4A and B). P-cadherin overexpression suppressed cell motility in DOK cells by ~50% (n=3). To determine if these results were, in part or whole, due to a difference in cell proliferation rates, we also analyzed the proliferative ability of control versus P-cadherin-overexpressing DOK lines using a thymidine nucleoside analog incorporation assay (Fig. 4C). This assay demonstrated no significant proliferation difference between control and P-cadherin-overexpressing cell lines.

### Both N- and P-cadherin expression are correlated with chemoresistance

The P-cadherin-induced mesenchymal characteristics we observed (Fig. 3) led us to examine the effect of P-cadherin overexpression on chemoresistance. In oral squamous carcinoma cells, resistance to 5-fluorouracil has been correlated with morphology and signaling changes characteristic of EMT 32. This medication is currently being utilized as part of a chemotherapy regimen for advanced oral squamous cell carcinoma 33. Because we found that P-cadherin overexpression increased N-cadherin protein levels in cells treated with 5-fluorouracil (Fig. 6B; described below), we also included a previously generated DOK line depleted of endogenous N-cadherin to distinguish P-cadherin-specific effects on cell survival.

Compared with the control cells, P-cadherin overexpression decreased apoptosis levels by ~2-fold at both 3 and 6 μM 5-fluorouracil concentrations (Fig. 5C). Conversely, depletion of endogenous N-cadherin increased apoptosis rates by 2- to 2.5-fold at the same concentrations compared with controls (Fig. 5C). Increased levels of either P- or N-cadherin therefore correlated with increased resistance to 5-fluorouracil-induced apoptosis. No statistically significant differences were found between P- or N-cadherin-altered cells and controls concerning necrosis (data not shown).

### P-cadherin-dependent alterations in epidermal-growth-factor-related signaling during 5-fluorouracil treatment

Because P-cadherin was previously shown to enhance EGF-induced Akt and MAPK signaling 17, we examined the effects of P-cadherin overexpression on EGFR-related signaling pathways in drug-treated DOK cells. In P-cadherin-overexpressing cells, EGFR showed strong membrane co-localization with exogenously expressed P-cadherin (Fig. 6A). Western blot analysis was used to characterize P-cadherin-dependent changes in EGFR-related signaling (Fig. 6B). Control and cadherin-altered DOK cells were treated with or without 3 µM 5-fluorouracil for 24 h before lysate preparation. Levels of cadherin proteins were also analyzed to account for alterations in expression caused by 5-fluorouracil treatment.

In cells treated with 5-fluorouracil, P-cadherin overexpression increased phosphorylation of Akt and MAPK compared to controls (Fig. 6B). P-cadherin overexpression also elevated N-cadherin protein levels in drug-treated cells. To assess the contribution of N-cadherin to these signaling changes, we examined the effect of 5-fluorouracil treatment in the N-cadherin-depleted line. Depletion of N-cadherin did not reduce downstream pathway activation; rather, phosphorylation of Akt and MAPK increased compared to controls (Fig. 6B). The ratio of Y1045-phosphorylated EGFR to total EGFR was decreased in P-cadherin-overexpressing cells under basal conditions. In drug-treated cells, N-cadherin depletion increased Y1045 phosphorylation, accompanied by reduced total EGFR levels (Fig. 6B).

### P-cadherin overexpression alters GSK3β and FAK levels

Because increased Akt signaling can promote EMT through inhibitory phosphorylation of GSK3β 34, levels of total and Ser9-phosphorylated GSK3β were also examined. In cells treated with 5-fluorouracil, the overexpression of P-cadherin resulted in increased levels of total GSK3β protein as well as GSK3β^Ser9^ (Fig. 7). Depletion of N-cadherin did not reduce GSK3β levels below control values (Fig. 7), indicating that endogenous N-cadherin in the dysplastic oral keratinocyte line was not responsible for the increases seen in P-cadherin-overexpressing cells.

Given the increased Akt and MAPK phosphorylation observed in P-cadherin-overexpressing cells (Fig. 6B), we also examined focal adhesion kinase (FAK), which can regulate both pathways 35. In P-cadherin-overexpressing DOK cells treated with 5-fluorouracil, levels of both phosphorylated FAK and total FAK increased compared to controls (Fig. 7). Similar increases were not observed in untreated cells.

## Discussion

We used the DOK cell line 18 as a model system to investigate the contributions of P-cadherin to tumor progression in oral potentially malignant disorders. Compared with HOK cells, the increased levels of P-cadherin observed in the DOK line are consistent with those reported in dysplastic human oral tissue 12, 13. The elevated N-cadherin levels observed in the DOK line may reflect a more advanced molecular phenotype, as N-cadherin upregulation has been more commonly associated with oral carcinomas 11. Levels of E-cadherin were comparable between the HOK and DOK lines, which is consistent with the retention of E-cadherin expression observed in healthy oral tissue and mild to moderate dysplasia 12, 13. The 3.6-fold increase in EGFR expression in the DOK line is also representative of the increased levels of EGFR seen in mildly dysplastic tissue 28. The elevation of proliferating cell nuclear antigen seen in the DOK line may reflect not only the proliferation rate of the cells but also an overexpression of PCNA that has been well-characterized in dysplastic epithelia 37.

Western blot data from this study suggest that the mesenchymal morphology seen in P-cadherin-overexpressing DOK cells may be mediated by increases in steady-state levels of the MUC1 protein. The C-terminal subunit of the MUC1 protein was shown to promote EMT through the activation of EMT-related transcription factors 29, 38, 39. The overexpression of P-cadherin was shown to increase motility in oral squamous carcinoma cells 17. In the dysplastic oral keratinocyte line, P-cadherin overexpression reduced motility by ~50%. These results are consistent with studies in breast and oral carcinoma cells, in which P-cadherin-dependent overexpression resulted in decreased migration 14, 40. Notably, P-cadherin overexpression did not alter E-cadherin levels in our study, indicating that these cells retained a key epithelial characteristic despite the acquisition of mesenchymal morphology.

The effects of P-cadherin overexpression in dysplastic oral keratinocytes differed markedly from those previously observed in the oral squamous carcinoma cell line SCC22A, in which P-cadherin increased motility but did not alter cell morphology or Akt signaling 17. Ribeiro and Paredes have proposed that P-cadherin expression may confer what Savagner referred to as an intermediate or metastable EMT state, in which cells retain some epithelial characteristics while gaining mesenchymal traits 8, 16. The DOK model demonstrates a similar pattern: P-cadherin-overexpressing cells acquired mesenchymal morphology and chemoresistance while retaining E-cadherin expression and suppressing motility. These findings suggest that the functional consequences of P-cadherin overexpression are stage-dependent, and that the acquisition of chemoresistance may occur independently of the migratory phenotype.

The increased expression of MUC1 may have contributed to chemoresistance in P-cadherin-overexpressing cells through multiple mechanisms. The highly glycosylated N-terminal subunit of MUC1 was shown to create a hydrophilic barrier at the outer cell membrane that could inhibit the uptake of chemotherapeutic agents, including 5-fluorouracil 41, 42*.* At the inner cell membrane, the MUC1 C-terminus was shown to bind to and enhance the signaling of the epidermal growth factor receptor, resulting in increased activity of the downstream modulators mitogen-activated protein kinase and Akt kinase 43-45. In the nucleus, the MUC1 C-terminus can bind separately to β-catenin 46, 47 or nuclear factor kappa B (NF-κB) 29, 48 to increase the transcription of genes involved in EMT. Both β-catenin and NF-κB activation have been shown by others to be essential modulators of chemoresistance to 5-fluorouracil 49, 50.

P-cadherin-mediated chemoresistance may also occur through signaling pathways that are independent of MUC1. In a breast carcinoma cell model, P-cadherin increased α6β4 integrin-mediated focal adhesion signaling that in turn increased the activity of FAK and Akt kinases 51. FAK has been shown to activate both Akt and MAPK signaling, as well as suppress apoptotic signaling pathways 35, 52. Consistent with these findings, we observed P-cadherin-dependent increases in activation of FAK, Akt and MAPK. These signaling changes may contribute to the chemoresistance observed in cadherin-altered DOK cells.

Elevated levels of either N- or P-cadherin correlated with increased chemoresistance in DOK cells. At 3 and 6 µM 5-fluorouracil, P-cadherin overexpression decreased apoptosis by ~2-fold, and cells with higher N-cadherin levels displayed a similar reduction in apoptosis compared with N-cadherin-depleted cells. Western blot analysis revealed both shared and distinct signaling features associated with P-cadherin compared to N-cadherin. Under basal conditions, P-cadherin overexpression decreased the ratio of Y1045-phosphorylated to total EGFR. Similarly, in drug-treated cells, those with higher N-cadherin levels displayed lower Y1045 phosphorylation and higher total EGFR levels than N-cadherin-depleted cells. Because Y1045 phosphorylation facilitates EGFR ubiquitination and degradation 53, these data suggest that both P- and N-cadherin may contribute to EGFR stability. In contrast, P-cadherin and N-cadherin showed opposing relationships with Akt and MAPK. P-cadherin overexpression increased phosphorylation of both kinases, whereas cells with higher N-cadherin levels displayed lower Akt and MAPK phosphorylation than N-cadherin-depleted cells. P-cadherin overexpression was also accompanied by increased FAK levels (Fig. 7), consistent with a previous report that P-cadherin activates FAK in basal-like breast carcinoma cells 51. Together, these observations suggest that P- and N-cadherin may influence chemoresistance through distinct mechanisms: P-cadherin-associated chemoresistance may involve FAK-related signaling 35, 52, whereas N-cadherin may modulate apoptotic signaling through decoy receptor interactions 54.

Taken together, these data suggest that P-cadherin overexpression during oral dysplasia facilitates a partial EMT in which cells acquire chemoresistance while retaining epithelial characteristics such as E-cadherin expression and reduced motility. In the context of field cancerization, the emergence of chemoresistant cells within dysplastic tissue prior to clinical detection of carcinoma could undermine both preventive and therapeutic interventions. Future studies using pharmacological inhibitors, rescue experiments, and MUC1 knockdown could clarify the mechanisms underlying these changes. Testing additional chemotherapeutic agents would also help define the drug specificity of this response, while work in primary dysplastic cells, patient-derived tissues, and in vivo models would help determine their relevance to human oral dysplasia. Such studies could establish P-cadherin as a promising therapeutic target for early intervention in oral potentially malignant disorders.

## Supplementary Material

Supplementary tables.

## Figures and Tables

**Figure 1:**
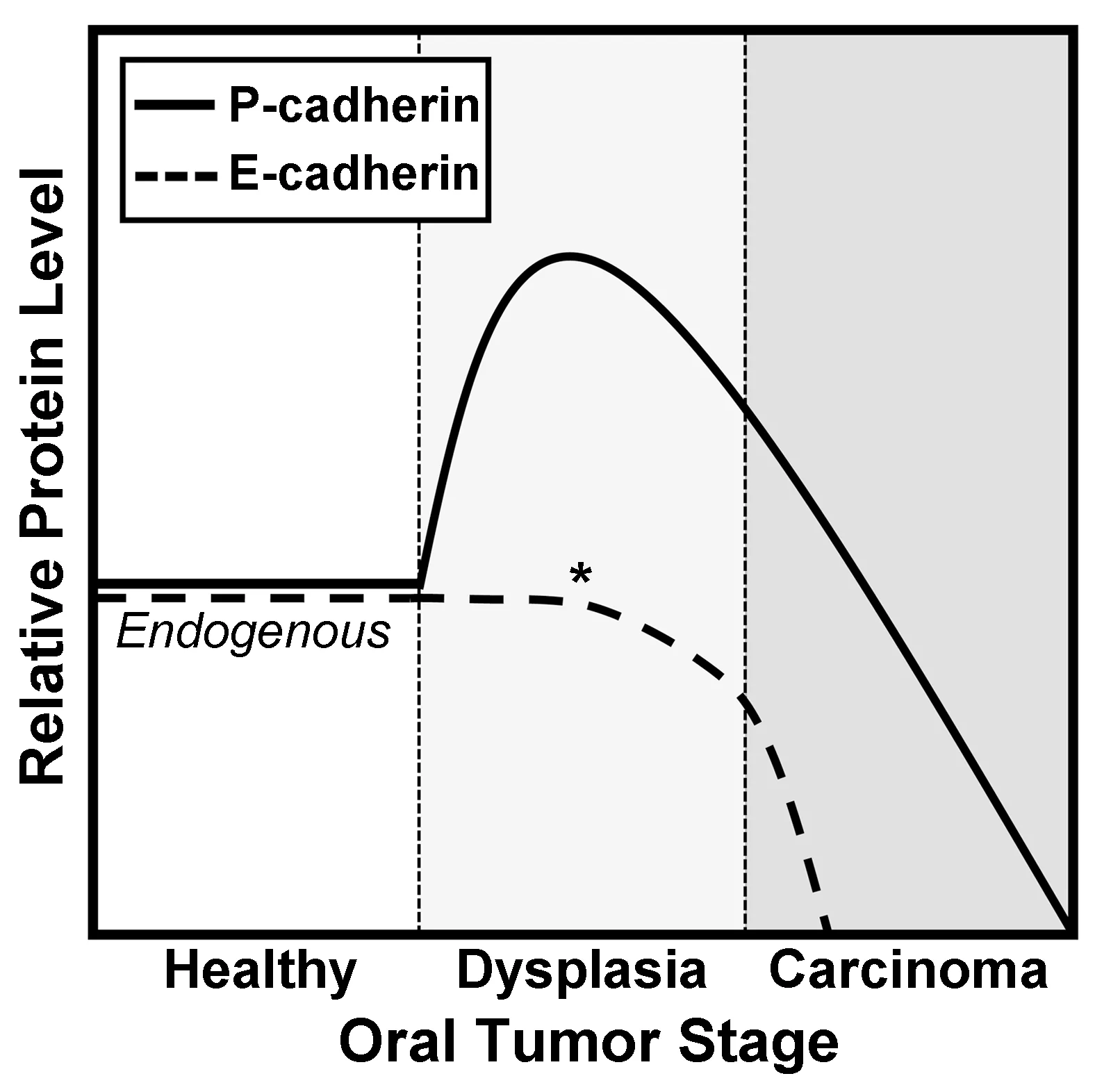
Model of classical cadherin expression patterns during oral tumor progression, based on published immunohistochemical studies. E-cadherin and P-cadherin are both components of healthy oral epithelia. During tumor progression, P-cadherin undergoes a uniquely transient increase in expression during mild to severe dysplasia and well-differentiated carcinomas 12, 13. P-cadherin expression is diminished or lost in late-stage carcinomas 14, 15. In contrast, E-cadherin levels remain comparable to normal epithelia until severe dysplasia and then diminish as tumors progress to carcinomas 12, 13. N-cadherin expression (denoted by an asterisk) has been reported in oral carcinomas 11 and in some dysplastic lesions 36.

**Figure 2:**
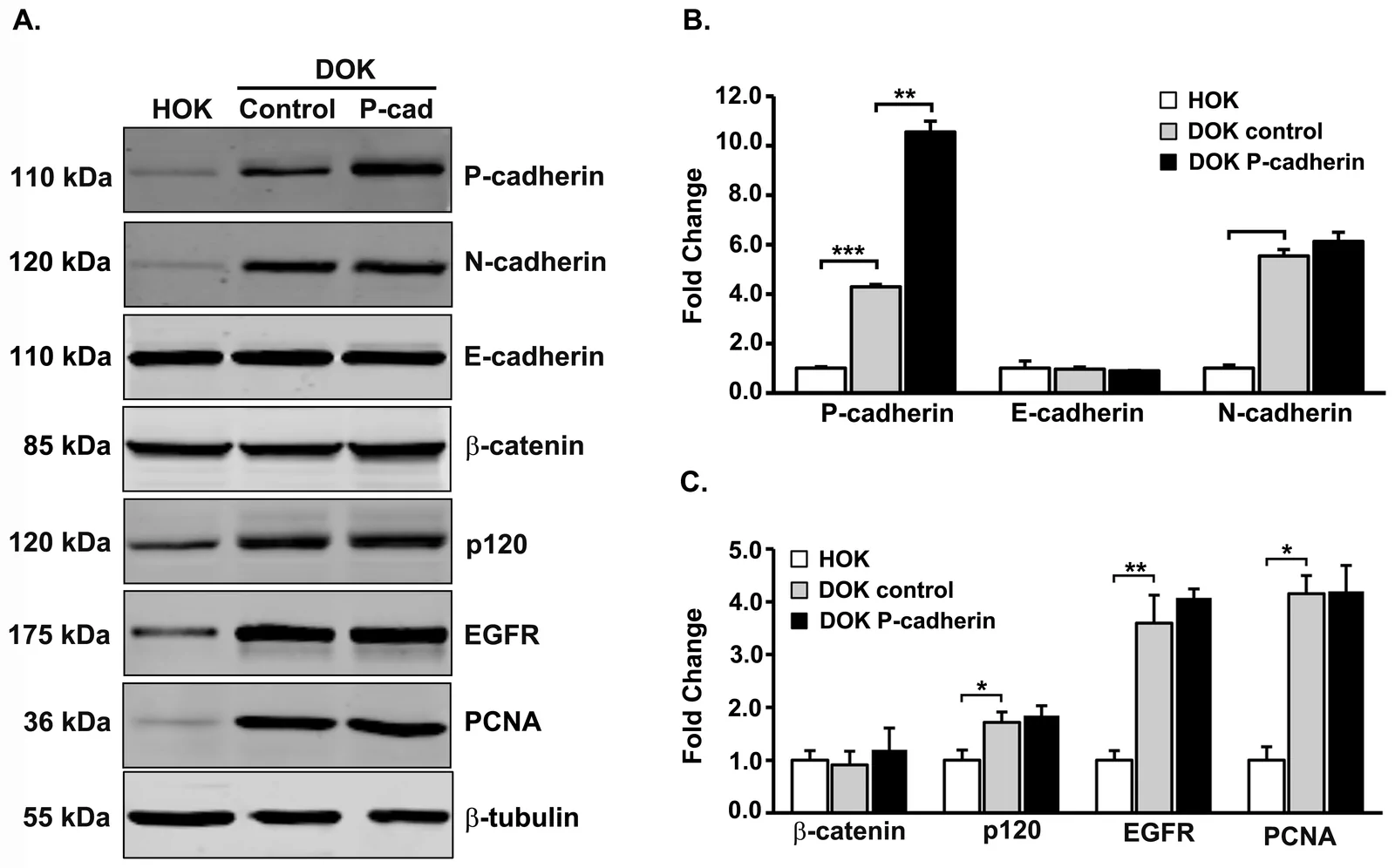
Comparison of adherens junction components and growth mediators in normal oral keratinocytes (HOK) and dysplastic oral keratinocytes (DOK). (A) Western blot analysis of adherens junction components and cadherin regulators (P-, E-, and N-cadherin, β-catenin, p120), epidermal growth factor receptor (EGFR), and proliferating cell nuclear antigen (PCNA). Lysates were prepared from primary HOK, DOK control and DOK cells expressing increased levels of P-cadherin (DOK P-cad). β-tubulin was included as a loading control. (B) Fold change in levels of P-cadherin, E-cadherin and N-cadherin analyzed in Fig. 2A. (C) Fold change in levels of β-catenin, p120, EGFR, and PCNA analyzed in Fig. 2A. For (B) and (C), fluorescence signal from each protein was normalized to total protein signal from the corresponding lane. Data are expressed as fold change compared to HOK band intensities. Data originated from three independent experiments. Error bars represent ± standard error of the mean. ^*^P≤0.05; ^**^P≤0.01; ^***^P≤0.001, using Student's two-tailed t-test.

**Figure 3:**
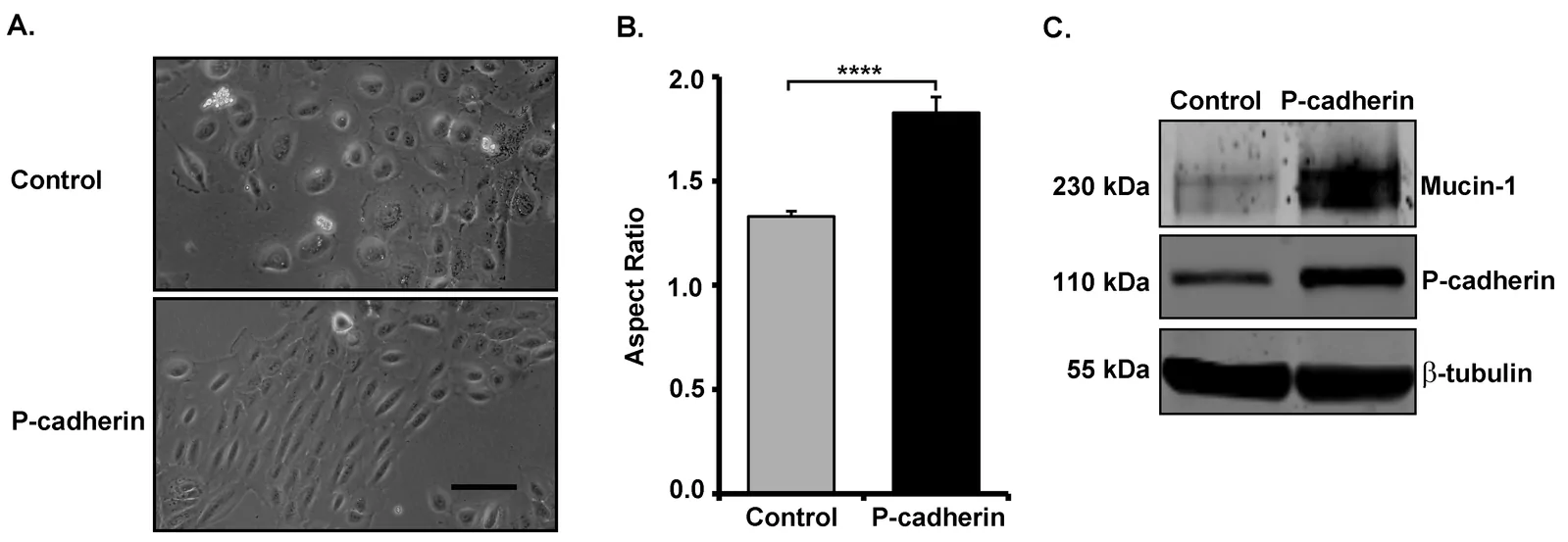
P-cadherin induces a mesenchymal morphology and increases expression of MUC1 protein. (A) Bright field photography of retrovirally transduced control and P-cadherin overexpressing DOK cells. Cells were photographed at x200 magnification, 24 h after plating. Scale bar, 100 µm. (B) Relative average aspect ratio (length/width) of control versus P-cadherin overexpressing DOK cells (n=75 cells per group, 25 cells from each of three independently plated dishes; ^****^P<0.0001). Error bars represent ± standard error of the mean. (C) Western blot analysis of MUC1 levels in control and P-cadherin overexpressing DOK cells.

**Figure 4:**
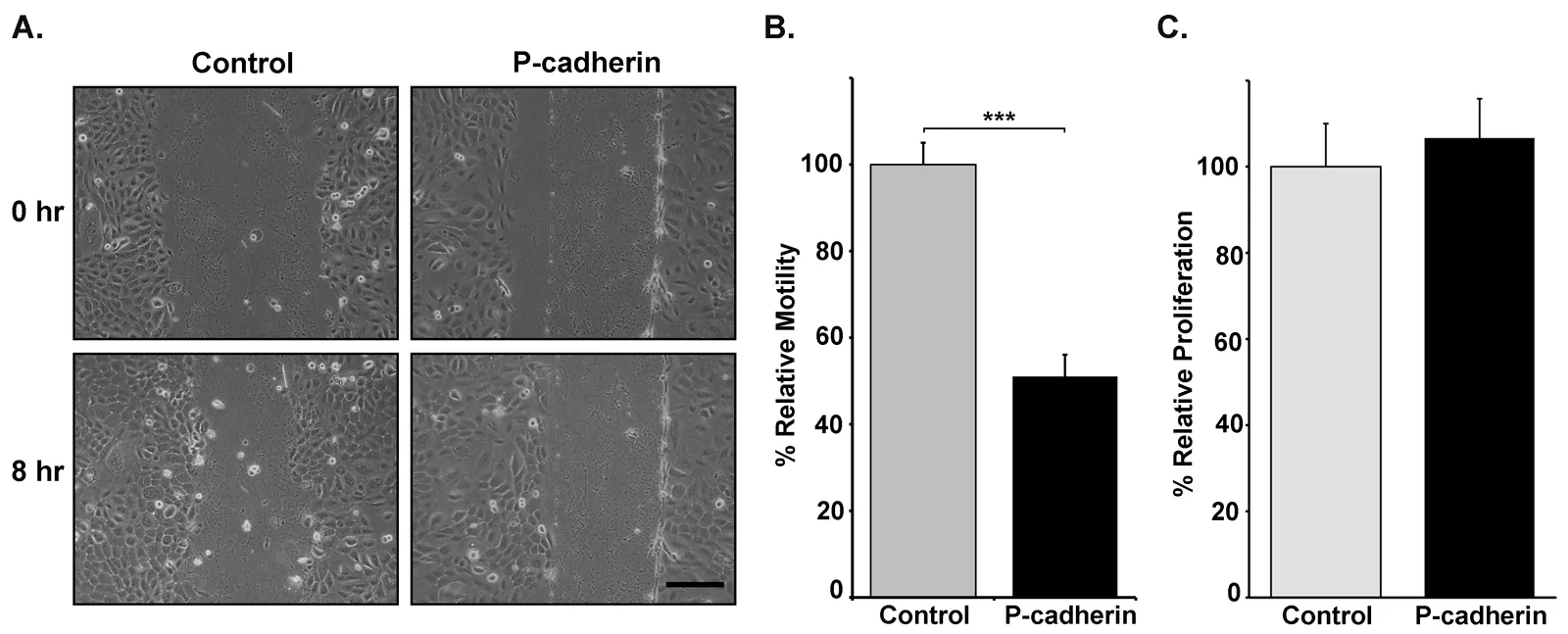
Effect of P-cadherin overexpression on motility and cell proliferation. (A and B) Wound healing analysis of DOK control and P-cadherin overexpressing cells. (A) Confluent monolayers were photographed at x100 magnification at the same location 0 and 8 h after wounding a confluent monolayer of a gridded dish with a pipet tip. Photographs are representative of control cells at 0 and 8 h, or P-cadherin-overexpressing cells at 0 and 8 h. Scale bar, 200 µm. (B) Relative motility of control and P-cadherin expressing cells. Data represent the mean of three independent experiments. ^***^P=0.00012 using Student's two-tailed t-test. (C) Proliferation of control and P-cadherin-overexpressing cells. Cells were plated at equal densities for 48 h and then labeled with the EdU thymidine analog for 24 h prior to fixation and fluorescence-based quantitation of signal. Data reflect the percentage of labeled cells in each population (n=8; P=0.42 using Student's two-tailed t-test). Error bars depict ± standard error of the mean.

**Figure 5:**
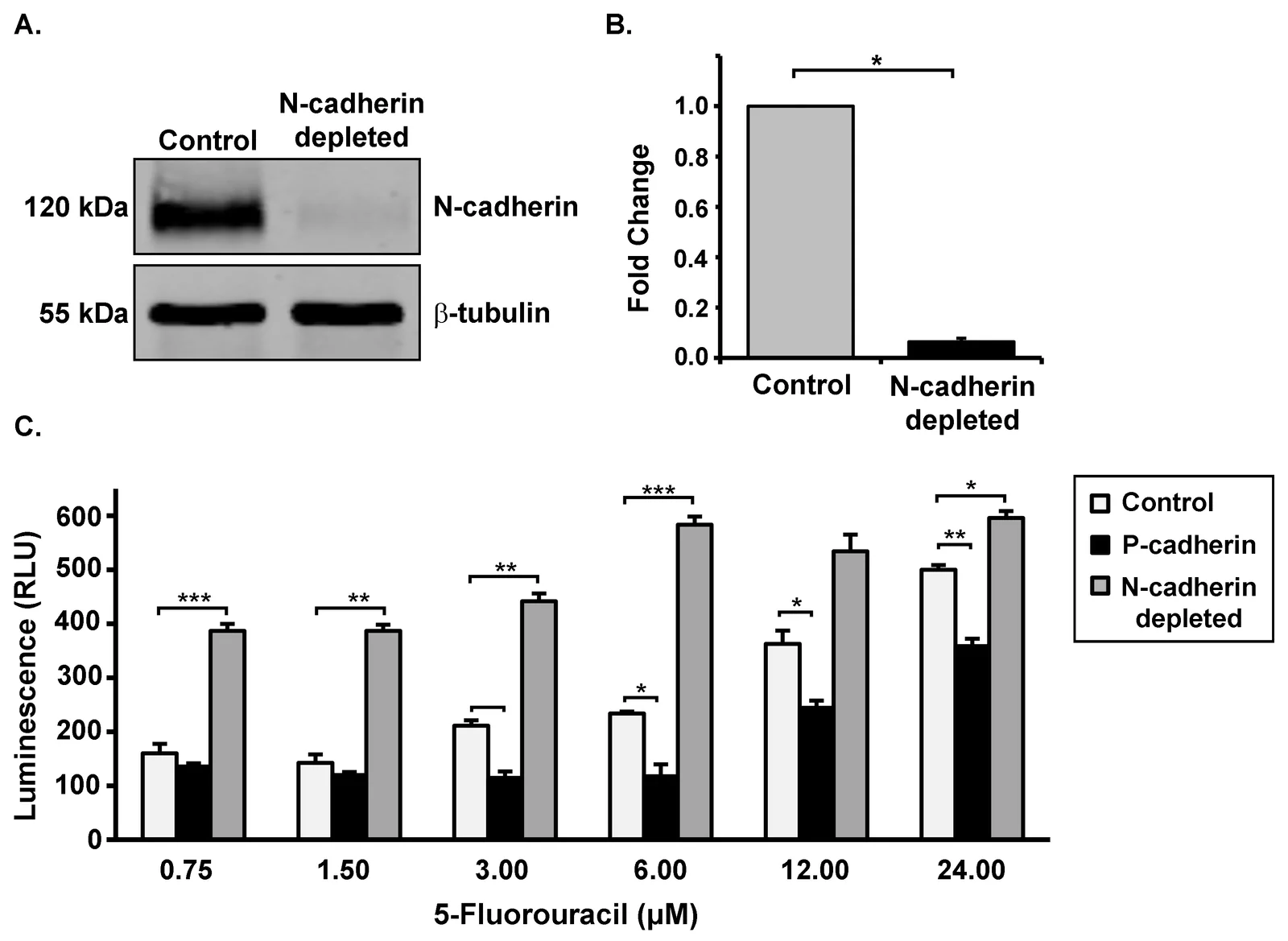
Depletion of N-cadherin and effect of P- and N-cadherin expression on apoptosis. (A) Western blot of DOK cells with depleted N-cadherin. Western blot analysis of lysates from control DOK cells and DOK cells depleted of N-cadherin by small hairpin RNA (N-depleted). Blots were probed for both N-cadherin and β-tubulin and imaged on the Odyssey CLX imaging system. (B) Quantitation of N-cadherin expression in control and N-cadherin-depleted DOK lines. For quantitation, the fluorescence signal from each N-cadherin signal was normalized to total protein signal from the corresponding lane. Data are expressed as fold change compared to control. Data originated from three independent biological replicates. Error bars represent ± standard error of the mean. ^*^P≤0.05. (C) Effect of altered P- and N-cadherin levels on apoptosis. DOK cells were plated for 48 h and then treated with 0, 0.75, 1.5, 3, 6, 12, and 24 µM 5-fluorouracil for 24 h. Apoptosis levels were measured at 0 and 24 h. The cells analyzed were the DOK control line (Control, white bars), DOK cells that overexpressed P-cadherin (P-cadherin, black bars), and DOK cells depleted of N-cadherin by small hairpin RNA (N-depleted, grey bars). Data originated from three independent biological replicates. Error bars at each data point depict ± standard error of the mean. ^*^P≤0.05, ^**^P≤0.01, ^***^P≤0.001, using Student's two-tailed t-test.

**Figure 6:**
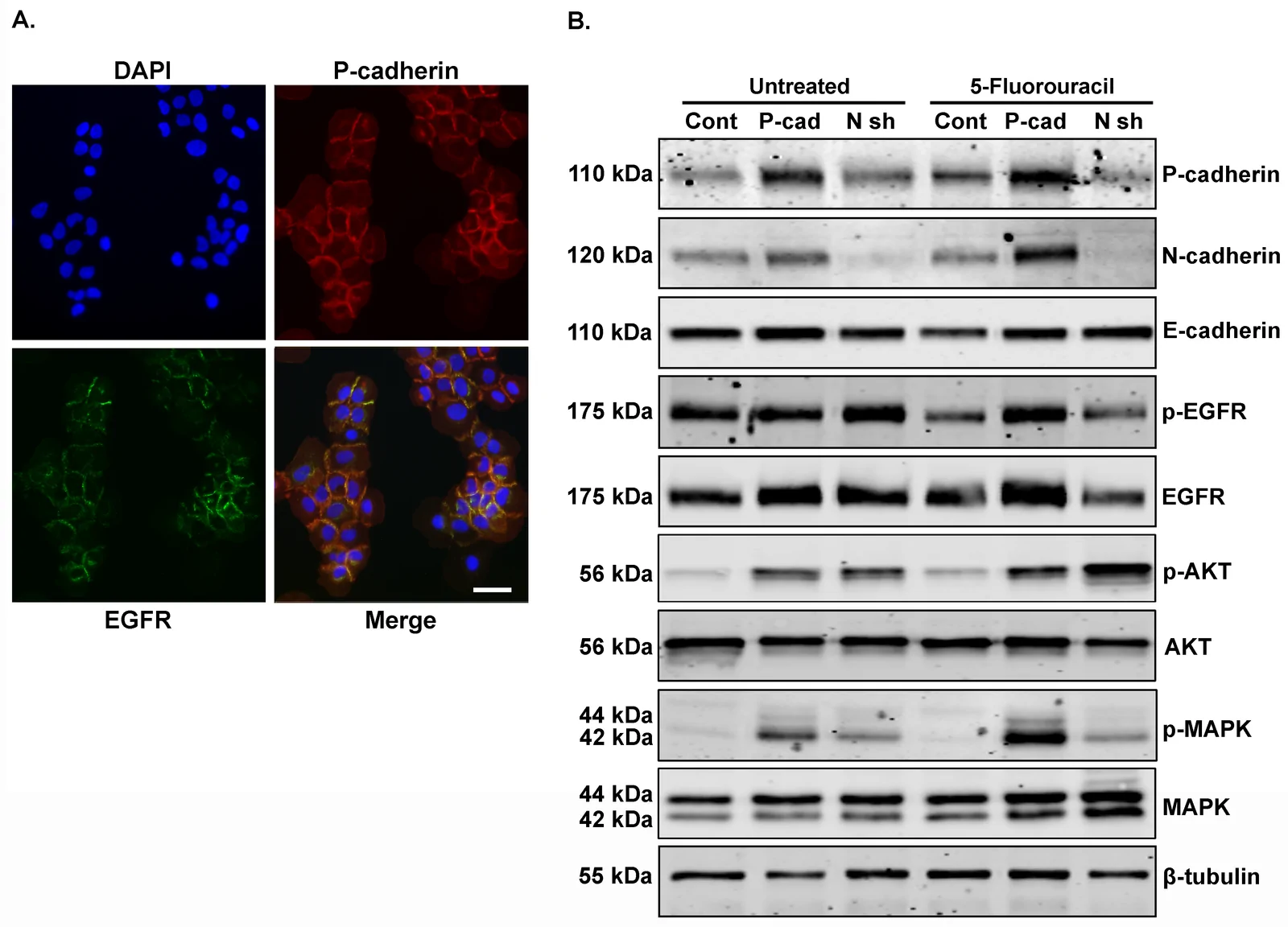
P-cadherin-dependent alterations in epidermal growth factor receptor-related signaling. (A) Co-localization of P-cadherin and epidermal growth factor receptor in P-cadherin-overexpressing DOK cells. Cells were plated at equal densities on glass cover slips and fixed 48 h after plating. After sequential incubation with P-cadherin and EGFR antibodies (and their Alexa 594- and 488-conjugated secondary antibodies, respectively), cells were mounted in DAPI-containing mounting media. AxioVision software was used to impart color to each image (blue, DAPI; red, P-cadherin; green, EGFR) and to create the composite image (Merge). Cells were photographed at x200 magnification. Scale bar, 50 µm. (B) Western blotting analysis of EGFR-related signaling proteins in cells treated with 5-fluorouracil. Control, P-cadherin-overexpressing, and N-cadherin-depleted DOK cells were treated with or without 3 µM 5-fluorouracil. Lysates prepared 24 h after sham or drug treatment were analyzed for P-cadherin, N-cadherin, E-cadherin, p-EGFR, total EGFR, p-Akt, total Akt, p-MAPK, and total MAPK. Levels of β-tubulin were utilized as a loading control. For phosphorylated proteins, the corresponding total protein was also detected as an additional loading control.

**Figure 7:**
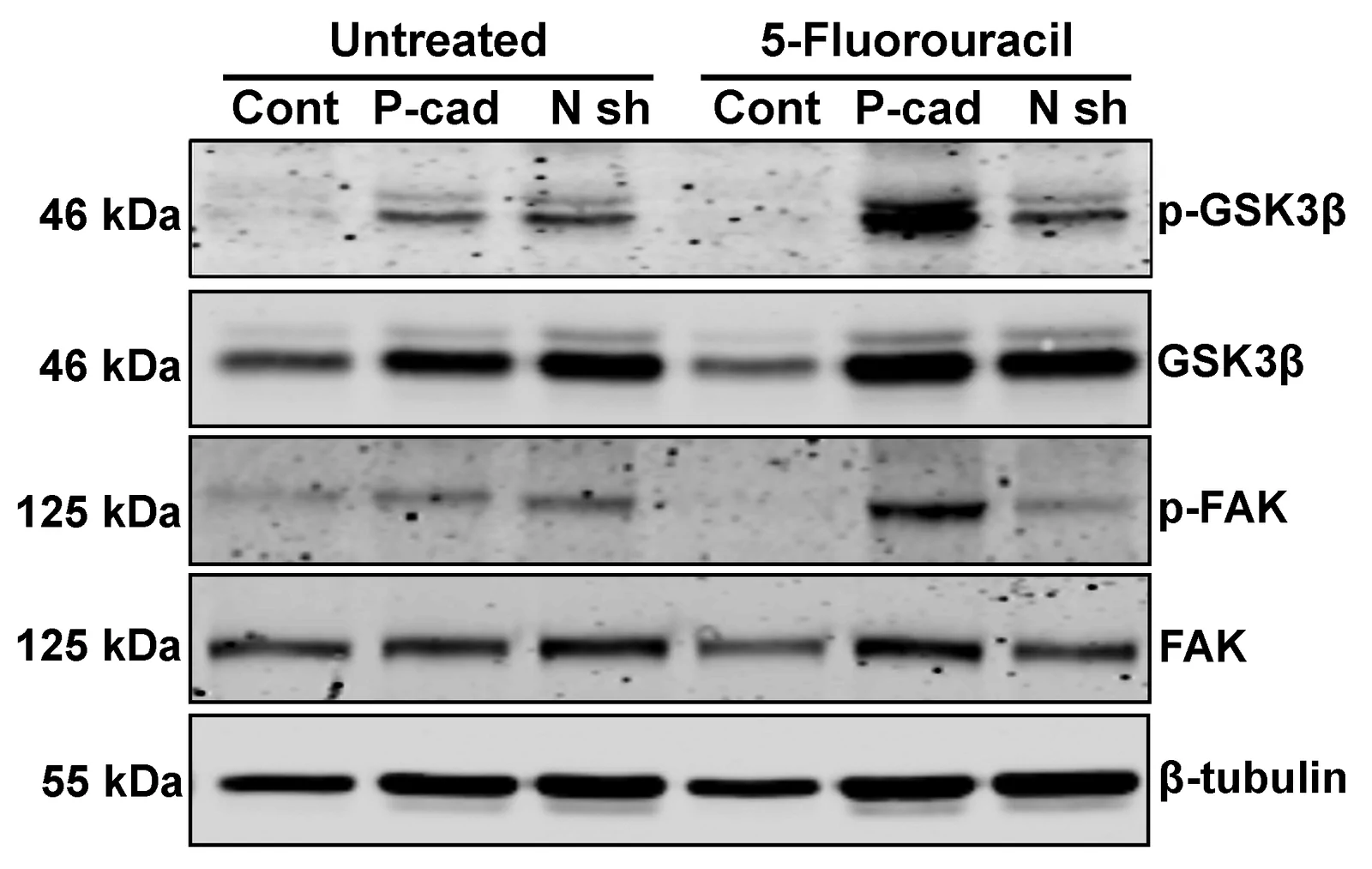
Survival signaling pathways modulated by P-cadherin. Lysates prepared from cells used in Fig. 6B were analyzed for p-GSK3β, total GSK3β, p-FAK, and total FAK. Levels of β-tubulin were utilized as a loading control. For phosphorylated proteins, the corresponding total protein was also detected as an additional loading control.

## Abbreviations

AoC: area of closure
DAPI: 4,6-diamidino-2-phenylindole
DMEM: Dulbecco's modified Eagle's medium
DOK: dysplastic oral keratinocytes
E-cadherin: epithelial cadherin
EdU: 5-ethynyl-2'-deoxyuridine
EGFR: epidermal growth factor receptor
EMT: epithelial-to-mesenchymal transition
FAK: focal adhesion kinase
GSK3β: glycogen synthase kinase 3β
HOK: human oral keratinocytes
MAPK: mitogen-activated protein kinase
MUC1: mucin-1
N-cadherin: neural cadherin
NF-κB: nuclear factor kappa B
p120: p120 catenin
PBS: phosphate-buffered saline
P-cadherin: placental cadherin
PCNA: proliferating cell nuclear antigen
RLU: relative luminescence units
SDS-PAGE: sodium dodecyl sulfate-polyacrylamide gel electrophoresis
