# Supplementary material for: P-cadherin Overexpression Induces Partial Epithelial-to-Mesenchymal Transition and Confers Chemoresistance in Dysplastic Oral Epithelial Cells
Source: J Cancer. 2026 Oct 16;17(11):1841–52. doi: 10.7150/jca.135818 (PMC13686773; doi:10.7150/jca.135818)
Supplement: Supplementary file 1 [file jcav17p1841s1.pdf]

Supplementary Table S1. Product information and weblinks for all cell lines and antibodies.

Material	Catalog No.	Manufacturer	Product Page URL
Cell Lines			
Human Oral Keratinocytes (HOK)	2610	ScienCell Research Laboratories	https://sciencellonline.com/en/human-oral-keratinocytes/
DOK (Dysplastic Oral Keratinocytes) RRID:CVCL_1180	94122104	Sigma-Aldrich (ECACC)	https://www.cellosaurus.org/CVCL_1180
Primary Antibodies (Mouse Host)			
P-cadherin (12H6)	14029	Cell Signaling Technology	https://www.cellsignal.com/products/14029
E-cadherin (4A2)	14472	Cell Signaling Technology	https://www.cellsignal.com/products/primary-antibodies/e-cadherin-4a2-mouse-monoclonal-antibody/14472
N-cadherin (13A9)	14215	Cell Signaling Technology	https://www.cellsignal.com/products/14215
β-catenin (15B8)	37447	Cell Signaling Technology	https://www.cellsignal.com/products/37447
p120 Catenin (98/pp120)	610134	BD Biosciences	https://www.bdbiosciences.com/en-us/products/reagents/microscopy-imaging-reagents/immunofluorescence-reagents/purified-mouse-anti-p120-catenin.610134
β-tubulin (E7)	E7	DSHB, University of Iowa	https://dshb.biology.uiowa.edu/E7_2
MUC1 (VU4H5)	4538	Cell Signaling Technology	https://www.cellsignal.com/products/primary-antibodies/muc1-vu4h5-mouse-monoclonal-antibody/4538
Primary Antibodies (Rabbit Host)			
Phospho-EGFR (Y1045)	2237	Cell Signaling Technology	https://www.cellsignal.com/products/2237
Total EGFR (D38B1)	4267	Cell Signaling Technology	https://www.cellsignal.com/products/4267
PCNA (D3H8P)	13110	Cell Signaling Technology	https://www.cellsignal.com/products/primary-antibodies/pcna-d3h8p-rabbit-monoclonal-antibody/13110
Phospho-p44/42 MAPK (T202/Y204) (D13.14.4E)	4370	Cell Signaling Technology	https://www.cellsignal.com/products/4370
Total p44/42 MAPK (137F5)	4695	Cell Signaling Technology	https://www.cellsignal.com/products/4695
Phospho-Akt (S473) (D9E)	4060	Cell Signaling Technology	https://www.cellsignal.com/products/4060
Total Akt (C67E7)	4691	Cell Signaling Technology	https://www.cellsignal.com/products/4691
Phospho-GSK-3β (S9) (5B3)	9323	Cell Signaling Technology	https://www.cellsignal.com/products/9323
Total GSK-3β (D5C5Z)	12456	Cell Signaling Technology	https://www.cellsignal.com/products/12456
Phospho-FAK (Y397) (D20B1)	8556	Cell Signaling Technology	https://www.cellsignal.com/products/8556
Total FAK (D5O7U)	71433	Cell Signaling Technology	https://www.cellsignal.com/products/71433
Secondary Antibodies (Western Blot)			
DyLight 680 Anti-mouse IgG	5470	Cell Signaling Technology	https://www.cellsignal.com/products/5470

Material	Catalog No.	Manufacturer	Product Page URL
DyLight 680 Anti-rabbit IgG	5366	Cell Signaling Technology	https://www.cellsignal.com/products/5366
DyLight 800 4X PEG Anti-mouse IgG	5257	Cell Signaling Technology	https://www.cellsignal.com/products/5257
DyLight 800 4X PEG Anti-rabbit IgG	5151	Cell Signaling Technology	https://www.cellsignal.com/products/5151
Secondary Antibodies (Immunofluorescence)			
Alexa Fluor 594 Anti-mouse IgG	A-11005	Thermo Fisher Scientific	https://www.thermofisher.com/order/catalog/product/A-11005
Alexa Fluor Plus 488 Anti-rabbit IgG	A32731	Thermo Fisher Scientific	https://www.thermofisher.com/order/catalog/product/A32731

All URLs were accessed September 2026.

Supplementary Table S2. Mean ± SD values for protein expression in HOK and DOK cell lines (Fig. 2B, 2C).

Figure 2B: Cadherins

Protein	HOK (Mean ± SD)	DOK Control (Mean ± SD)	DOK P-cadherin (Mean ± SD)	n
P-cadherin	1.00 (reference)	4.30 ± 0.15	10.56 ± 0.74	3
E-cadherin	1.00 (reference)	0.96 ± 0.15	0.90 ± 0.03	3
N-cadherin	1.00 (reference)	5.54 ± 0.46	6.13 ± 0.63	3

Figure 2C: Signaling mediators

Protein	HOK (Mean ± SD)	DOK Control (Mean ± SD)	DOK P-cadherin (Mean ± SD)	n
β-catenin	1.00 (reference)	0.91 ± 0.45	1.19 ± 0.72	3
p120	1.00 (reference)	1.72 ± 0.39	1.85 ± 0.37	3
EGFR	1.00 (reference)	3.60 ± 1.47	4.07 ± 1.60	3
PCNA	1.00 (reference)	4.15 ± 1.17	4.19 ± 1.67	3

Values represent fold change relative to HOK, normalized within each biological replicate.

Supplementary Table S3. Mean ± SD values for cellular phenotype analyses (Fig. 3B, 4B, 4C).

Measurement	DOK Control (Mean ± SD)	DOK P-cadherin (Mean ± SD)	n
Aspect ratio (Fig. 3B)	1.33 ± 0.26	1.83 ± 0.57	75
Relative motility (Fig. 4B)	100 ± 11	51 ± 11	3
Relative proliferation (Fig. 4C)	1.00 ± 0.07	0.94 ± 0.09	8

Motility and proliferation values are expressed relative to DOK control.

Supplementary Table S4. Mean ± SD values for N-cadherin expression and apoptosis assay (Fig. 5B, 5C).

Figure 5B: N-cadherin expression

Protein	DOK Control (Mean ± SD)	DOK N-cad depleted (Mean ± SD)	n
N-cadherin (fold change)	1.00 (reference)	0.06 ± 0.01	3

N-cadherin values represent fold change relative to control, normalized within each biological replicate.

Figure 5C: Apoptosis (5-fluorouracil dose response)

5-FU Concentration (μM)	DOK Control (Mean ± SD)	DOK P-cadherin (Mean ± SD)	DOK N-cad depleted (Mean ± SD)	n
0.75	160 ± 30	135 ± 10	386 ± 22	3
1.50	142 ± 27	119 ± 19	387 ± 19	3
3.00	211 ± 18	114 ± 21	442 ± 24	3
6.00	234 ± 6	117 ± 38	583 ± 26	3
12.00	363 ± 42	244 ± 16	534 ± 54	3
24.00	500 ± 15	358 ± 23	596 ± 22	3

Values reported as relative luminescence units (RLU).
